# Thoracic spine pain in the general population: Prevalence, incidence and associated factors in children, adolescents and adults. A systematic review

**DOI:** 10.1186/1471-2474-10-77

**Published:** 2009-06-29

**Authors:** Andrew M Briggs, Anne J Smith, Leon M Straker, Peter Bragge

**Affiliations:** 1 School of Physiotherapy and Curtin Health Innovation Research Institute, Curtin University of Technology, GPO Box U1987, Perth, 6845, Western Australia, Australia; 2 Global Evidence Mapping (GEM) Initiative, Department of Surgery (Royal Melbourne Hospital), University of Melbourne, Parkville 3010 VIC, Australia

## Abstract

**Background:**

Thoracic spine pain (TSP) is experienced across the lifespan by healthy individuals and is a common presentation in primary healthcare clinical practice. However, the epidemiological characteristics of TSP are not well documented compared to neck and low back pain. A rigorous evaluation of the prevalence, incidence, correlates and risk factors needs to be undertaken in order for epidemiologic data to be meaningfully used to develop evidence-based prevention and treatment recommendations for TSP.

**Methods:**

A systematic review method was followed to report the evidence describing prevalence, incidence, associated factors and risk factors for TSP among the general population. Nine electronic databases were systematically searched to identify studies that reported either prevalence, incidence, associated factors (cross-sectional study) or risk factors (prospective study) for TSP in healthy children, adolescents or adults. Studies were evaluated for level of evidence and method quality.

**Results:**

Of the 1389 studies identified in the literature, 33 met the inclusion criteria for this systematic review. The mean (SD) quality score (out of 15) for the included studies was 10.5 (2.0). TSP prevalence data ranged from 4.0–72.0% (point), 0.5–51.4% (7-day), 1.4–34.8% (1-month), 4.8–7.0% (3-month), 3.5–34.8% (1-year) and 15.6–19.5% (lifetime). TSP prevalence varied according to the operational definition of TSP. Prevalence for any TSP ranged from 0.5–23.0%, 15.8–34.8%, 15.0–27.5% and 12.0–31.2% for 7-day, 1-month, 1-year and lifetime periods, respectively. TSP associated with backpack use varied from 6.0–72.0% and 22.9–51.4% for point and 7-day periods, respectively. TSP interfering with school or leisure ranged from 3.5–9.7% for 1-year prevalence. Generally, studies reported a higher prevalence for TSP in child and adolescent populations, and particularly for females. The 1 month, 6 month, 1 year and 25 year incidences were 0–0.9%, 10.3%, 3.8–35.3% and 9.8% respectively. TSP was significantly associated with: concurrent musculoskeletal pain; growth and physical; lifestyle and social; backpack; postural; psychological; and environmental factors. Risk factors identified for TSP in adolescents included age (being older) and poorer mental health.

**Conclusion:**

TSP is a common condition in the general population. While there is some evidence for biopsychosocial associations it is limited and further prospectively designed research is required to inform prevention and management strategies.

## Background

Spinal pain is a well recognised condition associated with significant personal and community burdens. The most common spinal regions studied are the lumbar and cervical spine, probably because of their strong and well-established associations with pain conditions, work-related injuries, intervertebral disc degenerative conditions, headaches and psychosocial disturbances [1,2]. Compared to the lumbar and cervical spine, the thoracic spine has received less attention in terms of clinical, genetic and epidemiologic research [3,4], yet pain experienced in the thoracic spine can be equally disabling, imposing similar burdens on the individual, community [4-6] and workforce [7]. In the context of this paper, we refer to thoracic spine pain (TSP) as pain experienced in the region of the thoracic spine, between the boundaries of T1–T12 and across the posterior aspect of the trunk. TSP may arise from a number of sources including thoracic and cervical spinal structures, the thorax, and the gastrointestinal, cardiopulmonary and renal systems [3,8,9]. Moreover, the thoracic spine is a common site for inflammatory, degenerative, metabolic, infective and neoplastic conditions which may also contribute to pain and disability [10].

The limited research on prevalence and risk factors for TSP likely reflects the belief that the clinical and public health significance of TSP is less compared to other spinal levels. Nonetheless, it has been argued that TSP should be considered as a discrete and important clinical entity, independent of pain experienced in other areas of the spine [11], and particularly in youth where TSP is common, disabling and has an increasing incidence with age during adolescence [11,12]. There is also evidence to suggest that pain or dysfunction in the thoracic spine is not trivial in adulthood [13]. Alarmingly, preliminary evidence suggests the incidence of spinal pain among otherwise healthy adolescents is increasing, which may suggest a new and expanding condition burden for future adults [14]. More international studies are required to verify whether an increasing incidence is a global phenomenon. This highlights the importance of examining modifiable risk and prognostic factors for spinal pain, including TSP, from childhood to adulthood.

TSP and dysfunction are associated with conditions such as primary and secondary osteoporosis, particularly vertebral fractures [15-18] and hyperkyphosis arising from vertebral bone loss [19], ankylosing spondylitis [20], osteoarthritis [21] and Scheuermann's disease [22]. However, little attention has been paid to TSP among individuals who have no history of a metabolic, inflammatory or structural disorder, despite such non-specific, mechanical TSP being a common presentation in clinical practice [23]. Similar to the lumbar spine, degenerative signs identified in the thoracic spine with imaging modalities are not necessarily associated with pain, suggesting that non-specific TSP is also prevalent [3,24].

Understanding the prevalence and risk factors of TSP in the otherwise healthy general population is important for several reasons. Firstly, musculoskeletal dysfunction in this population is likely to impose a significant community burden, particularly when considering reduced productivity in young-adult and middle-aged working populations. Secondly, interpretation of TSP in disease needs to be evaluated against normative population-based data. Thirdly, such information can be harnessed to develop evidence-based preventative and treatment strategies for this condition. Emerging evidence from cross-sectional and prospective cohort studies, as cited in this paper, suggests that TSP is prevalent among healthy individuals and does impact on function [4,11,13], yet to our knowledge no reviews have been published which evaluate and synthesise these data from childhood to adulthood. Although an earlier review reported the prevalence and incidence of idiopathic TSP in youth, no data were included on adult cohorts nor were any correlates or risk factors for TSP reported, a small number of databases were searched, and the included studies were not critically appraised [25]. Here, we extend the findings of that review by including adults and address the abovementioned limitations. A major limitation of previous research is the use of a combined outcome measure for spinal pain. That is, specific results for the thoracic spine are rarely reported. Rather, only low back or just 'back' pain is reported which may encompass more than one spinal area. A similar limitation has also been identified in an earlier systematic review of neck pain [26], while other authors argue that a standardised definition of spinal pain is urgently needed which specifies time period recall, symptoms and anatomical areas [27-29]. Therefore, the aim of this review was to systematically review and report evidence describing the prevalence, incidence, associations (cross-sectional study) and risk factors (prospective study) for TSP in the general population outside the context of late adulthood and who are free of other pathology.

## Methods

A systematic review method was used to address the aim of this study. Systematic reviews use explicit search, study selection and appraisal methods to address a focused clinical question [30]. Systematic reviews are less prone to bias than narrative reviews, in which non-systematic approaches to searching, selection and appraisal are employed [31]. The structure and content of this systematic review complies with recommendations outlined by the Meta-analysis of Observational Studies in Epidemiology (MOOSE) group for reporting meta-analyses of observational studies [32].

### Data sources and searches

Nine databases (Medline, CINAHL, PubMed, ISI Web of Science, PEDro, EMBASE, Cochrane, AMED, BioMed Central) were searched using search strings listed in Appendix 1, from inception to January 2008. Automatic search alerts were set up in each database to alert the authors to any new papers published which met the search criteria between January 2008 and February 2009; however, no further papers were identified during this period. In addition, reference lists of included papers were searched to identify other potentially suitable studies. Shorter and simpler search strings were used for databases that did not use subject headings or that had a limited number of allowable search terms (Cochrane, PEDro, BioMed Central). Search strings pertaining to prevalence and risk factors were based on a previously conducted systematic review of prevalence and risk factors for musculoskeletal disorders [33]. In addition, keywords were mapped to subject headings (MeSH headings) in MEDLINE to identify synonyms for 'epidemiological', 'thoracic spine' and 'musculoskeletal disorder' terms.

### Study selection

For studies to be included in this review, the following criteria had to be met:

1. The cohort (children, adolescents or adults) had to be community-based so that cohorts studied were population-based, rather than specific to certain occupational, clinical, or athletic groups. For example, studies which reported TSP characteristics among a cohort of individuals with osteoporosis or other musculoskeletal pathologies or diagnosed structural deformities (e.g. scoliosis) were excluded. Therefore only idiopathic presentations of TSP in the general population were included.

2. The study had to report *either *prevalence, incidence, associated factors, or risk factors for thoracic spine pain specifically (cervico-thoracic and thoraco-lumbar were also accepted). The outcome variables could be self-reported or clinically evaluated. Any self-reported pain experienced in the thoracic spine, dorsal spine, upper back or mid-back was accepted and no inclusion criteria were imposed pertaining to pain severity, frequency, duration or pain-related disability as there are no agreed criteria for these in the context of TSP.

3. The study design had to be case-control, cross-sectional or cohort (prospective-cohort or retrospective-cohort). Case-control and cross-sectional studies are appropriate for investigating prevalence and correlates, while prospective or retrospective cohort studies are appropriate for investigating incidence and risk factors [34,35].

4. The study had to be published in a peer-reviewed journal in English.

**Figure 1:**
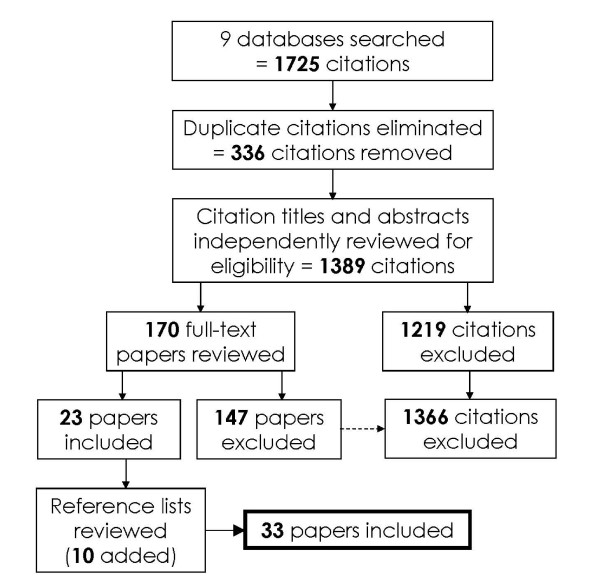
**Flowchart illustrating process for systematic review and assessing inclusion criteria**.

Titles and abstracts of citations were assessed for inclusion eligibility by two independent reviewers (AB, AS), both experienced musculoskeletal science researchers. Full text articles appearing to meet the above criteria were retrieved and evaluated against the inclusion criteria. Full text articles were also retrieved and evaluated in circumstances where the abstract was not available, or if it was not clear whether the article met the inclusion criteria for the review based on the content of the abstract. Disagreement regarding eligibility for inclusion, at the level of both title/abstract and full text review, was resolved by a consensus meeting between the authors. Figure 1 illustrates the systematic review process for this paper.

### Data extraction and quality assessment

Study quality was assessed using two methods by two independent reviewers (AB, AS). First, the study design of each eligible study was ranked using the revised Australian National Health and Medical Research Council (NHMRC) Hierarchy of Evidence framework [35] (Appendix 2). We considered this hierarchy to be appropriate as it comprises levels of evidence for each type of research question (intervention, diagnostic accuracy, prognosis, aetiology, screening intervention). For this review, we considered the hierarchy of evidence for 'aetiology' to be the most appropriate category. Hierarchical ranking provides a broad indication of the methodological strength of a study.

Second, an in-depth appraisal of method quality was conducted using the Critical Review Form – Quantitative Studies [36]. This appraisal tool evaluates method rigor and bias using a combination of dichotomous (yes/no) and descriptive items. The descriptive items are listed at the foot of Additional file 1. The decision to select a yes/no score was based on the experience of the raters, instructions accompanying the tool, and applicability of the domains relative to the design of the study being appraised. Any disagreements in dichotomous scores between the independent reviewers were resolved by consensus. An arbitrary quality score was obtained by sum-totalling 15 relevant dichotomous quality appraisal criteria in this tool, with a score of 1 indicating fulfilment of the criterion and a score of 0 indicating non-fulfilment or non-description of the criterion. Thus, a higher score represented higher method quality. This critical appraisal tool was chosen because:

a) It evaluates the domains 'appropriate selection of participants' and 'appropriate measurement of variables'. These domains have been recommended as fundamental to an observational epidemiological critical appraisal tool, given that there is no consensus in the literature regarding a 'gold standard' appraisal tool for observational epidemiological studies [37].

b) It can be used for a variety of study designs, including epidemiological studies, as reflected by previously published epidemiological systematic reviews that have employed this tool [33,38]. The use of a generic, rather than a design-specific critical appraisal tool facilitates comparison of methodological quality items common to different study designs (such as reliability and validity of outcome measurement) across the included studies.

c) Detailed instructions for use are provided, facilitating consistency in their interpretation and application [38].

### Data synthesis and analysis

The following data were extracted by the chief author (AB): cohort characteristics including ethnicity, participant numbers and gender, age; mode of TSP data collection; prevalence and incidence of TSP as percentages; associations (cross-sectional studies) and risk factors (prospective studies) for TSP as either odds ratios, correlation co-efficients, chi square, regression, or Mann Whitney U-test statistics, depending on the analysis methods used in the source papers. Where data were presented in a Figure, the corresponding author of the paper was contacted and asked to provide the dataset. In circumstances where the data set was not available, data were interpolated from the Figure. Results of the included studies were narratively synthesised.

## Results

### Searching and inclusion

Of the 1389 citations retrieved from the 9 databases searched (Appendix 3), 23 (1.7%) [11-13,39-58] were selected for review on the basis of meeting the inclusion criteria (Figure 1). 1366 (98.3%) papers were excluded because TSP was not reported specifically. A further 10 papers were selected after reviewing reference lists of the included papers [59-68]. Therefore, 33 papers were included in the review.

### Study design and quality assessment

Additional file 1 summarises the study design, cohort characteristics, method of TSP data collection, hierarchy of evidence score for studies of aetiology, and quality scores for each study.

Of the 33 papers included in the review, 26 (78.8%) were cross-sectional surveys (NHMRC evidence level IV), 5 (15.2%) were prospective cohort studies (NHMRC evidence level II) and 2 (6.0%) were retrospective cohort studies (NHMRC evidence level III-2) [35] (refer to Appendix 2 for definitions of NHMRC ranks).

The mean (SD) quality score from evaluation of study quality using the Critical Review Form – Quantitative Studies [36] was 10.5 (2.0) out of 15. The most common method flaws identified according to the quality assessment tool were sampling biases (inadequate blinding for physical measures, response rates below 80%), inadequate sample size justification (power calculations), lack of detail regarding informed consent and a lack of information regarding the reliability and validity of outcome measures used. On the other hand, all studies used an appropriate design to address the proposed research question and the vast majority reported data in terms of statistical significance (e.g. reporting a 95% confidence interval for odds ratios or a *p*-value for correlation co-efficient), commented on the clinical importance of the findings, used appropriate analysis methods (ie the correct statistical test), reached appropriate conclusions given their results reported (i.e. did not comment on issues for which there were no data to support the claims), and the outcomes offered implications for clinical practice (Additional file 1).

### Data extraction and synthesis

The majority of cohorts were European (n = 25, 75.7%), while Canadian/USA (n = 4, 12.1%), New Zealand/Australian (n = 2, 6.1%) and Asian (n = 2, 6.1%) populations were less commonly studied.

Additional file 2 summaries the prevalence and incidence data for TSP across age groups according to the operational definition of TSP used in each study. There was considerable variability in the operational definitions of TSP employed in the various studies. This contributed to the large prevalence and incidence ranges across ages. In particular, the point prevalence ranged from 4–72% with the lower limit derived from a study where TSP was defined as "any back pain" while the upper derived from a study where TSP was defined as "pain associated with backpack use". A similar situation was noted for 7-day and 1-year prevalence. Of the 33 studies, 19 (57.6%) employed a pain definition as "any pain", while 4 (12.1%) used "pain while carrying a backpack", 1 (3.0%) used "pain interfering with school or leisure activities", 8 (24.2%) defined pain with a specific duration and/or frequency and 1 (3.0%) used "pain after work". TSP prevalence also varied with age. For example, the 1-month prevalence ranged from 1.4% in adults aged 40–69 years to 34.8% in children aged 12 years.

**Figure 2:**
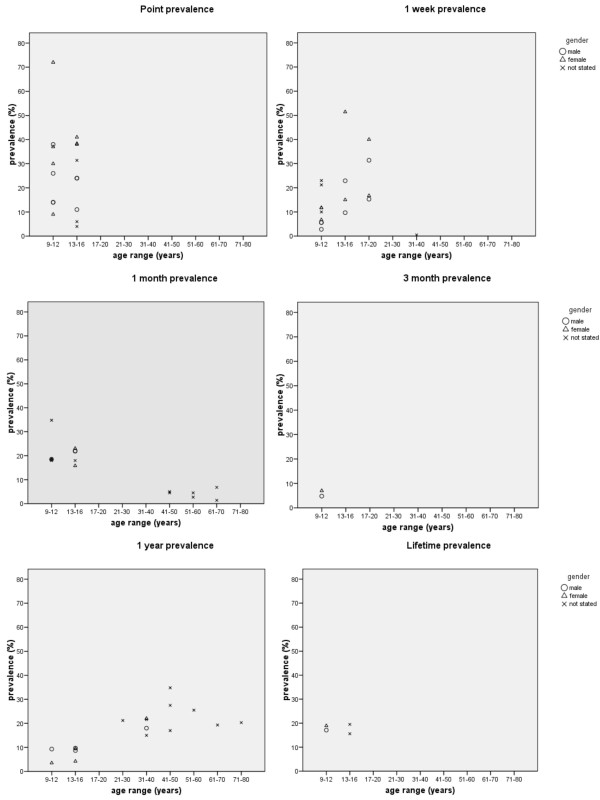
**Period prevalence data expressed by age range and gender**. Data are not reported for prevalence of cervicothoracic and thoracolumbar pain (4 studies). In studies where an age range was reported, the median age was calculated for the 'age range' variable. Studies reporting only a minimum age for inclusion were excluded from the Figure [51,56]. Therefore, the Figure shows data from five studies which reported point prevalence, 6 for 1-week prevalence, 6 for 1-month prevalence, 1 for 3-month prevalence, 6 for 1-year prevalence, and 3 for lifetime prevalence.

There were 31 reports of TSP prevalence in 29 studies across 6 prevalence periods. There were 5 (16.1%) reports of point prevalence, 8 (25.8%) 7-day prevalence, 6 (19.4%) 1-month prevalence, 1 (3.2%) 3-month prevalence, 7 (22.6%) 1-year prevalence and 4 (12.9%) lifetime prevalence. Generally, studies reported a higher prevalence for TSP in child and adolescent populations, and particularly for females. These data are summarised in Figure 2. There were 6 reports of TSP incidence in 5 studies across 5 incidence periods. Similarly, there was marked variability in the 1-year incidence data (3.8–35.3%) which likely reflects differences in age and pain definitions between the studies. Generally, incidence of TSP was higher among females, other than at the ages of 16 and 17 where the incidence of TSP in adolescent boys was greater than girls in one study [12].

Additional file 3 summarises all associated and risk factors reported for TSP across 15 studies according to 7 biopsychosocial categories. Across the individual studies, TSP was significantly associated with concurrent musculoskeletal pain and, growth and physical, lifestyle and social, backpack, postural, psychological, and environmental factors. The majority of studies (n = 13, 85.7%) were cross-sectional in design, providing only evidence of association between factors and TSP. Two prospective studies established significant risks factor for the development of TSP. Having poorer mental health [69] and being an older compared to younger adolescent [12] were identified as risk factors for TSP in adolescence.

## Discussion

To our knowledge this is the first systematic review of the prevalence, incidence, associated factors and risk factors for TSP among the general population. We elected to study and report all these epidemiologic characteristics to present a comprehensive picture of what is known about TSP in the general population. Findings are consistent with clinical anecdotes suggesting that TSP is common in the general population, particularly during adolescence, yet interpretation of the data are difficult due to heterogeneity. Despite the large ranges identified in TSP prevalence, predominantly as a result of variability in definitions of pain, these data support the contention that TSP is prevalent among youth. Whether it should be considered as a discrete and important clinical presentation in youth and perhaps treated as such [11], would depend on how often TSP coexists with other spinal pain conditions. Conversely, in adulthood there are insufficient data to draw a similar conclusion. Unfortunately, the data reviewed do not provide comprehensive information about the impact of TSP on function. Nonetheless, up to 10% of adolescents experienced TSP that interfered with school or leisure [45] and TSP prevalence seemed to be highest during backpack use. Although some data have been collected regarding associated and risk factors for the condition, these are relatively scarce, highlighting the need for further epidemiologic research directed towards this condition. Most importantly, a consistent approach with respect to defining pain characteristics and reporting prevalence and incidence data is urgently needed among researchers to allow meaningful comparisons between studies.

### Study design and quality assessment

The NHMRC supports a 4-point rating scale (excellent, good, satisfactory, poor) for each of the 5 essential components of a body of evidence: evidence base, consistency of results, clinical impact, generalisability, and applicability [35]. The majority of studies included in this review were cross-sectional in design, limiting inferences about causality and prognosis for TSP. Thus, in terms of the evidence base reviewed (relating primarily to study design), it may be rated as poor according to NHMRC criteria. Prospective cohort studies are therefore required to provide a more robust evidence base for prognostic factors and the clinical course of the condition across the lifespan. Moreover, these studies would also provide important information to clinicians regarding the natural history of TSP and ultimately trajectories in certain clinical groups. Nonetheless, we suggest that the evidence presented is satisfactory with respect to consistency, clinical impact, generalisability and applicability. Generally, the method quality of the included studies was good, with only one older study being rated particularly low (4/15) [42]. More than 90% of the studies reviewed used an appropriate study design, used appropriate analysis methods, reported results in terms of statistical significance, provided a commentary on the clinical relevance, and reached appropriate conclusions given the results presented. The most significant method quality issue identified was a lack of sample size justification, with only 2 (6.1%) studies addressing this criterion with a power calculation. However, in observational studies concerning prevalence and incidence a sample size estimate is not as critical as in intervention studies, since demonstrating a difference between groups is rarely needed. We acknowledge, however, that power is needed in order to detect relationships in epidemiologic studies. The independent reviewers also identified biases in many studies, primarily related to sampling bias. We considered a sample bias to be present if the response rate to a questionnaire was less than 80% [70]. Finally, only 33.3% and 42.4% of the included studies presented evidence about the validity and reliability, respectively, of the outcome measures used. The comprehensive quality assessments performed in this study highlight areas for improvement in research design and reporting in the context of spinal pain.

### TSP prevalence

The range of prevalence estimates of TSP in the general population was broad. Similarly, a broad range of TSP prevalence was reported in a review of TSP among adult working populations [7]. The wide prevalence range is partially a reflection of the influence of age and gender. However, even within an age range and gender category, prevalence estimates were highly variable. For example, point prevalence in children ranged from 14–38% among males (based upon 2 studies) and 9–72% among females (2 studies). This may result from the variable operational definitions or study inclusion criteria for pain cases in cross-sectional studies. Notably, the studies on young people were often focussed on pain related to school bags and workstations which may also have influenced the reported rates. The operational definition issue has been identified as a major limitation in the comparability between prevalence studies in low back pain research [29].

Variability in operational definitions of TSP may also influence the interpretation of TSP across prevalence periods. As highlighted in Figure 2, children had higher TSP prevalence than adults for one month prevalence, while the reverse was observed for one year prevalence. This inconsistency may partly be explained by recall period, where children are less likely to recall events over a longer duration. However, a more likely explanation may be the differences in operational definitions of TSP between studies. Data for one month prevalence of TSP in youth was sourced from four studies [11,50,57,67] while only one study was available for adult data [55]. The operational definition for TSP in the adult study was "frequent pain in the upper back" compared to "any pain" or "pain duration ≥ 1 day" in the youth studies. Fewer adults were likely to report frequent pain as compared to definitions that were unrelated to pain frequency. Similarly, for the one year prevalence, six adult studies contributed to the data and adopted a definition of "any pain" [13,46,47,68], "pain duration for ≥ 1 week" [51], or "pain after work" [39], while one youth study reported a definition of "pain interfering with school or leisure" [45]. The lower prevalence in the youth study was likely related to a definition of pain which needed to be associated with a functional impairment.

To assess the effect of study quality on the prevalence ranges we excluded the 8 studies which scored less than the mean method-quality score (< 10/15). Excluding these studies had a minimal effect on prevalence other than raising the lower limit of 7-day prevalence to 2.8% (from 0.5%), raising the lower limit of 1-year prevalence to 15.0% (from 3.5%), and leaving one study reporting a lifetime prevalence of 15.6% (from 15.6–19.5%). This finding is consistent with an earlier study which reported prevalence estimates of neck pain to be unrelated to study quality [71]. We did not perform a similar sensitivity analysis based on NHMRC Hierarchy of Evidence rank since the majority of studies were ranked as level IV, thus over representing this type of study design relative to others.

We identified 6 prevalence periods and 4 incidence periods in the literature for TSP. Prevalence data were distributed relatively equally across the 6 periods, other than the 3 month period where only one report of TSP prevalence was made in one study [66]. Therefore, it seems that there is not only a lack of consensus with respect to definitions of pain and inclusion criteria between studies, but also the most appropriate prevalence period to investigate. The 7-day and 1-year prevalence periods were the most commonly reported (25.8% and 22.6% respectively), consistent with an earlier systematic review of neck pain [71]. Moreover, they are also consistent with recall periods in the Nordic Musculoskeletal Pain Questionnaire [72], which is one of the most commonly used assessment tools in musculoskeletal research. Although recall bias may be more problematic with longer recall periods (e.g. 1-year) [73], shorter time frames (e.g. 7-day) may miss episodes of pain. In light of evidence which supports the validity of recalling pain intensity for at least a 3-month recall period [29], we suggest that for chronic and disabling spinal pain, recall bias is less likely to be threatened. Nevertheless, such variability in definitions renders interpretation of the data somewhat difficult, and this issue has been highlighted previously as a limitation in the comparability of spinal pain research [27-29,71]. A recent international Delphi study concluded that definitions for prevalence studies on low back pain should include, at a minimum, the site of low back pain, symptoms observed, time frame of the measure, and severity [29]. Arguably, these same criteria should be applied to TSP studies. Consistent with the consensus of an international working party for low back pain research [29], we recommend a 1 month prevalence period for studies investigating TSP.

Similar to findings in this review, prevalence estimates for low back and neck pain vary widely in the literature. Among the general adult population, the point, 12-month and lifetime prevalence for low back pain ranges from 5.6–28.4%, 22–65%, and 11–84% across various studies [46,74-76], while for neck pain these estimates are 5.9–38.7%, 16.7–75.1%, and 0.2–71% across various studies [71,77] and for TSP the 12-month prevalence ranges from 15–34.8% [13,46,47,51,68]. Notwithstanding the wide prevalence ranges and variability of spinal pain definition, it appears that TSP may be a significant a problem in adulthood. In adolescents the point, 12-month and lifetime prevalence for low back pain ranges from 1.0–35.8%, 7.0–50.8%, and 7.0–72.0% respectively [25] and for neck pain the 12-month and lifetime prevalence range from 7.6–13.0% and 3.0–28.0% respectively [25]. In this review the point, 12-month and lifetime for TSP in adolescents (13–20 years) was reported to range from 4.0–41.0%, 4.2–9.7%, and 15.6–19.5% suggesting comparable significance to low back and neck pain in adolescents. Notably, the point prevalence for TSP in children was even higher (4.0–72.0%), suggesting the magnitude of the problem of TSP, in terms of prevalence, to be greatest in youth. This may be one reason why there are a greater number of studies using childhood and adolescent cohorts compared to adult cohorts. However, further interpretation of the impact of TSP should be made with due consideration to the severity and disability associated with the experience of TSP.

Higher TSP prevalence in females is consistent with general reports of musculoskeletal pain in adults [78], adolescents [79,80] and children [81]. A higher prevalence of self-reported pain among females may be due to differences in physical activity, musculoskeletal maturity, posture, endocrine and psychosocial characteristics as well as different physiological mechanisms for pain perception between genders [82], which should be investigated.

### TSP Incidence

Interpretation of incidence data is limited due to the small number of studies that report TSP incidence. The method quality of these studies was generally high (range: 9/15–13/15). Excluding the one study with a quality score below the mean did not significantly change the interpretation of TSP incidence other than excluding the report of 25-year incidence [62]. However, as with prevalence data, there was considerable variability in the pain definitions and incidence periods, and females generally experienced a higher incidence of TSP, except during late adolescence.

### TSP burden

Unlike neck and low back pain, the burden of TSP has not been well established, which represents an important avenue for future research. Within a cohort of Danish children and adolescents, TSP was the most commonly reported site of spinal pain and 38% of the cohort reported some kind of impact from spinal pain, such as reduced physical activity and care-seeking [11]. Similarly, in a cohort of adults reporting TSP, 23.5% reported difficulty with activities of daily living due to pain (compared to 30.3% and 41.1% for neck and low back pain respectively) while the median (IQR) for the number of days where pain was experienced during activities of daily living was 13.5 (5.0–30.0) for TSP, 7.0 (3.0–30.0) for neck pain and 10.0 (4.0–30.0) for low back pain [13]. Moreover, TSP has been identified as a significant predictor of failure of returning to work in good health among individuals who present with back pain in primary care [23]. Collectively, these data suggest that TSP imparts an impact comparable to neck and low back pain.

### Associated factors and risk factors for TSP

In children and adolescents, TSP was associated with female gender, postural changes associated with backpack use, backpack weight, other musculoskeletal symptoms, participation in specific sports, chair height at school, and difficulty with homework, while poorer mental health and age transition from early to late adolescence were significant risk factors for TSP. In adults TSP was associated with concurrent musculoskeletal symptoms and difficulty in performing activities of daily living and there were no studies reporting risk factors. Although the limited data describing the associated and risk factors for TSP established predominantly with bivariate analyses render interpretation of its aetiology difficult, the factors identified in this review suggest that musculoskeletal growth, biomechanical loading, concurrent musculoskeletal pain and psychosocial characteristics are important mediators. Therefore, a biopsychosocial framework would seem appropriate for conceptualising TSP aetiology among the general population who are free of other pathology.

### Strengths and limitations

Strengths of this review include a systematic review method, in particular the use of an appropriate critical appraisal tool for observational epidemiological literature. Additionally, tailoring of the search strategy, via the use of broad search terms, was employed to capture studies that reported on the prevalence, incidence or risk of TSP even where these parameters were not the study's primary objective. This search approach formed the rationale of an earlier review [71]. However, the findings presented here should be interpreted within the limitations of the review. Firstly, the studies included were generally from high income countries and therefore the data reported may not represent TSP from a global perspective [83]. Future studies should examine whether any differences exist in TSP experiences between ethnic groups. Secondly, studies published in languages other than English were not reviewed. Thirdly, studies reporting epidemiologic data for TSP in discrete occupational groups were not included, as risk factors for spinal pain would likely be influenced by, and differ between occupations and not be representative of the general population. Finally, studies in this review involving children and adolescents were derived from samples of schoolchildren and therefore do not represent children who do not attend school, for example those involved in child labour [84]

## Conclusion

The information presented in this systematic review confirms a relatively high prevalence of TSP in the general population and substantiate the view that TSP is a discrete and important clinical condition. Considering the high reported prevalence estimates for TSP and relatively scarce information concerning risk factors, further research should be directed towards the epidemiology of TSP, particularly in adolescence, using prospective cohort designs. Careful consideration should be given to minimising sampling bias and using valid and reliable outcome measures. Furthermore, it will be important for future studies to use a consistent and appropriate definition of TSP, and specifically avoid using simply 'back pain' as an outcome variable, a recommendation endorsed by a recent international consensus [29]. Moreover, an exploration of prognostic factors should include psychological, physical, occupational and social mediators of TSP [85].

## Competing interests

The authors declare that they have no competing interests.

## Authors' contributions

AB developed the research question, coordinated the review, contributed to the research design, performed the literature search, performed data extraction, and contributed to study selection, quality appraisal and manuscript writing. AS contributed to study selection, quality appraisal and manuscript writing. LS contributed to the research question and manuscript writing. PB contributed to the research question, research design, quality appraisal and manuscript writing. All authors read and approved the final manuscript.

## Authors' information

AB is a clinical physiotherapist and NHMRC postdoctoral research fellow. AS is an NHMRC postdoctoral research fellow and physiotherapist. LS is a professor, NHMRC senior research fellow and physiotherapist. PB is a research fellow and physiotherapist.

## Appendix 1

Search strings used in electronic databases. The * symbol indicates truncation for the search term.

### Population

child* or adult* or worker* or adolescent* or schoolchild* or student* or profession* or pediatric or paediatric

AND

### Location

thoracic spine or dorsal spine or mid* back or upper back or thoracolumbar or cervicothoracic

AND

### Condition

pain or discomfort or back pain or musculoskeletal disorder* or dysfunction or disability or disabilities or musculoskeletal disease or injur* or occupational disease or occupational disorder

AND

### Study

causality or cohort stud* or cross-sectional stud* or epidemiolog* or epidemiologic factor* or follow-up study or incidence or incidence studies or prevalence or prevalence studies or prospective studies or risk or risk factor or survey

### Limits

English language, human studies, fields (title or abstract)

### Exclusion terms (NOT)

surgery or surgical or operative or fracture or osteoporosis

#### Complete search strategy using * truncation

(child* OR adult* OR worker* OR adolescent* OR schoolchild* OR student* OR profession* OR pediatric OR paediatric) AND (thoracic spine OR dorsal spine OR mid* back OR upper back OR thoracolumbar OR cervicothoracic) AND (pain OR discomfort or back pain OR musculoskeletal disorder* OR dysfunction OR disability OR disabilities OR musculoskeletal disease OR injur* OR occupational disease OR occupational disorder) AND (causality OR cohort stud* OR cross-sectional stud* OR epidemiolog* OR epidemiologic factor* OR follow-up study OR incidence OR incidence studies OR prevalence OR prevalence studies OR prospective studies OR risk OR risk factor OR survey) NOT (surgery OR surgical OR operative OR fracture* OR osteoporo*)

#### Complete search strategy for PubMed

(child OR children OR childhood OR adult OR adults OR adulthood OR worker* OR adolescent* OR schoolchild* OR student* OR profession* OR pediatric* OR paediatric* OR pediatrics) AND (thoracic spine OR dorsal spine OR "mid back" OR "midback" OR "middle back" OR "upper back" OR thoracolumbar OR cervicothoracic) AND (pain OR discomfort OR back pain OR back ache OR backache OR musculoskeletal disorder* OR dysfunction OR disability OR disabilities OR musculoskeletal disease OR injury OR injuries OR injured OR occupational disease OR occupational disorder*) AND (causality OR cohort stud* OR cross-sectional stud* OR epidemiolog* OR epidemiologic factor* OR follow-up study OR incidence OR incidence studies OR prevalence OR prevalence studies OR prospective studies OR risk OR risk factor OR survey OR surveys) NOT (surgery OR surgical OR operative OR fracture* OR osteoporo* OR surgical procedures, operative)

### Abridged search strings

Thoracic spine or dorsal spine or mid$ back or upper back

AND

Pain or disorder* or injur*

## Appendix 2

NHMRC Evidence Hierarchy: designations of levels of evidence according to Aetiologyresearch questions.

### Level – Descriptor

I – A systematic review of level II studies

II – A prospective cohort study

III-1 – All or none*

III-2 – A retrospective cohort study

III-3 – A case-control study

IV – A cross-sectional study or case series

* all or none of the people with the risk factor(s) experience the outcome; the data arises from an unselected or representative case-series which provides an unbiased representation of the prognostic effect.

The complete levels of evidence document may be viewed at 

## Appendix 3

Database search results

### Database – First search date – Citations

Medline – 8-Jan-08 – 174

CINAHL – 8-Jan-08 – 760

PubMed – 9-Jan-08 – 211

ISI Web of Science – 8-Jan-08 – 443

BioMed Central – 9-Jan-08 – 4

PEDro – 8-Jan-08 – 64

EMBASE – 9-Jan-08 – 53

Cochrane – 8-Jan-08 – 1

AMED – 8-Jan-08 – 15

SUM = 1725

Duplication = – 336

Citations for review = 1389

## Pre-publication history

The pre-publication history for this paper can be accessed here:



## Supplementary Material

Additional File 1 **Cohort characteristics, TSP data source, hierarchy of evidence score and quality appraisal for each study**. The data provided describe the characteristics of each paper included in the review and results of the quality appraisal. Click here for file

Additional File 2 **Prevalence (29 studies) and incidence (5 studies) data grouped by pain definition and reported by age**. The data provided describe the prevalence and incidence of TSP across the included studies. Click here for file

Additional File 3 **Associated (cross-sectional studies) and risk (prospective studies) factors for TSP grouped according to biopsychosocial categories. Estimates are expressed as an odds ratio (95% CI) unless indicated otherwise. An OR > 1 refers to a positive association with TSP, while and OR < 1 refers to a negative association. Where correlation coefficients are reported, positive values represent a positive association with TSP, while negative values represent a negative association. For statistical tests which do not test for association, a significant outcome (p < 0.05) refers to the factor being greater among individuals with TSP**. The data provided summarise factors associated with, and risk factors for TSP. Click here for file
