# Supplementary material for: Thoracic spine pain in the general population: Prevalence, incidence and associated factors in children, adolescents and adults. A systematic review
Source: BMC Musculoskelet Disord. 2009 Jun 29;10:77. doi: 10.1186/1471-2474-10-77 (PMC2720379; doi:10.1186/1471-2474-10-77)
Supplement: Additional File 1 — Cohort characteristics, TSP data source, hierarchy of evidence score and quality appraisal for each study. The data provided describe the characteristics of each paper included in the review and results of the quality appraisal. [file 1471-2474-10-77-S1.pdf]

Additional File 1 Cohort characteristics, TSP data source, hierarchy of evidence score and quality appraisal for each study.

Reference	Population	TSP data source	N	Age (yrs)	NHMRC score	Quality appraisal score*															
						1	2	3	4	5	6	7	8	9	10	11	12	13	14	15	Total
Aronsson et al [39]	Swedish working adults	telephone interview	2086 F, 1715 M	16-65	IV	1	1	1	1	0	1	0	1	1	1	1	1	1	1	0	12
Balague et al [60]	Swiss schoolchildren	self-report questionnaire	875 F, 840 M	7-17	IV	1	1	1	1	0	0	0	0	0	1	1	1	1	0	0	9
Balague et al [61]	Swiss schoolchildren	self-report questionnaire	323 F, 292 M	12-17	IV	1	0	1	0	0	0	0	1	1	1	1	1	1	1	1	10
Balague et al [59]	Swiss schoolchildren	self-report questionnaire	54 F, 63 M	10-16	IV	1	0	1	0	0	1	1	0	1	1	1	1	1	1	0	10
Cude-Mauroux et al [40]	Swiss schoolchildren	self-report questionnaire	68 F, 57 M	8-12	IV	1	1	1	1	1	1	1	0	1	1	1	1	1	1	1	14
Dieck et al [62]	American college graduates	self-report questionnaire	903 F	Not stated	III-2	1	0	1	0	0	0	0	0	1	1	1	1	1	1	1	9
El-Metwally et al [41]	Finnish schoolchildren	self-report questionnaire	571 F, 542 M	10.8	II	1	1	1	1	0	1	0	1	1	1	1	1	1	1	1	13
Fairbank et al [42]	UK schoolchildren	self-report questionnaire	219 F, 227 M	12-18	IV	0	0	1	0	0	0	0	0	0	1	0	1	0	1	0	4

		and subjective interview																			
Feldman et al [69]	Canadian high school students	self-report questionnaire	342	13.8 (0.1)	II	1	1	1	0	0	1	1	0	0	1	1	1	1	1	1	11
Grimmer et al [12]	Australian schoolchildren	subjective interview	60 F, 72 M	13-17	II	1	1	1	1	0	1	1	0	0	1	1	1	1	1	1	12
Hagen et al [43]	Norwegian adults	self-report questionnaire	25,922	20-80	IV	1	1	1	0	0	1	0	0	0	1	1	1	1	1	1	10
Harreby et al [64]	Danish schoolchildren	self-report questionnaire	718 F, 671 B	14-15	IV	1	0	1	0	0	0	0	0	0	1	1	1	1	1	0	7
Korovessis et al [44]	Greek schoolchildren	subjective interview	664 F, 588 M	15.0 (2.0)	IV	1	0	1	0	0	1	1	0	1	1	1	1	1	1	0	10
Korovessis et al [65]	Greek schoolchildren	subjective interview	1816 F, 1625 M	12.0 (1.5)	IV	1	0	1	0	0	1	1	0	1	1	1	1	1	1	1	11
Kujala et al [45]	Finnish schoolchildren	self-report questionnaire	344 F, 354 M	10-16	IV	1	0	1	0	0	1	0	1	0	1	1	1	1	1	0	9
Linton et al [47]	Swedish adults	self-report questionnaire	1004 F, 910 M	35-45	IV	1	1	1	1	0	0	0	0	0	0	1	1	1	1	0	8
Linton et al [46]	Swedish adults	self-report questionnaire	500	35-45	IV	1	1	1	0	0	1	0	0	1	1	1	1	1	1	0	10
Mikkelsson et al [66]	Finnish schoolchildren	self-report questionnaire	894 F, 862 M	9.8 (0.3) and	II	1	1	1	1	0	1	0	1	1	1	1	1	1	1	1	13

				11.8 (0.4)																	
Mogensen et al [67]	Danish schoolchildren	subjective interview	234 F, 205 M	12-13	IV	1	1	1	1	0	1	1	1	0	1	1	1	1	1	1	13
Moore et al [48]	American schoolchildren	subjective interview	287 F, 244 M	8-18	IV	1	1	1	0	0	1	1	0	0	1	1	1	1	1	1	11
Murphy et al [49]	UK schoolchildren	self-report questionnaire	32 F, 34 F	12.7 (0.9)	IV	1	1	1	0	0	1	1	0	0	1	1	1	1	1	1	11
Murphy et al [50]	UK schoolchildren	self-report questionnaire	336 F, 343 M	12.8 (0.9)	IV	1	1	1	0	0	1	1	1	1	1	1	1	1	1	1	13
Natvig et al [68]	Norwegian adults	self-report questionnaire	1418 F, 1308 M	20-72	IV	1	1	1	0	0	1	0	1	1	0	1	0	1	1	1	10
Niemelainen et al [13]	Finnish males	structured interview	600 M	49.8 (7.7)	IV	1	1	1	0	0	1	1	0	1	1	1	1	1	1	1	12
Park et al [51]	American working male adults	self-report questionnaire	12,812	>19 yrs	III-2	1	1	1	1	0	1	1	0	0	1	0	1	1	0	1	10
Reigo et al [52]	Swedish working adults	self-report questionnaire	663 F, 681 M	20-59	IV	1	0	1	0	0	1	0	1	1	1	0	1	1	1	0	9
Salminen et al [53]	Finnish schoolchildren	self-report questionnaire	370	11-17	IV	1	1	1	0	0	1	0	1	0	1	1	1	1	1	1	11
Troussier et al [54]	French schoolchildren	self-report questionnaire	471 F, 455 M	10-14	IV	1	0	1	0	0	1	0	0	0	1	1	1	1	1	1	9
Tsuritani et	Japanese	subjective	709 F	54.0	IV	1	1	1	1	0	1	0	0	0	1	1	1	1	1	0	10

al [55]	female adults	interview		(7.7)																	
Veerapen et al [56]	Adults living in Malaysia	subjective interview	1420 F, 1174 M	> 15	IV	1	0	1	0	0	1	0	0	0	1	1	1	1	1	1	9
Wedderkopp et al [57]	Danish female schoolchildren	subjective interview	254 F children , 165 F adolescents	8-10, 14-16	IV	1	1	1	1	0	1	0	0	0	1	1	1	1	1	1	12
Wedderkopp et al [11]	Danish schoolchildren	subjective interview	419 F, 387 M	8-16	IV	1	0	1	0	1	1	1	1	0	1	1	1	1	1	1	12
Whittfield et al [58]	New Zealand schoolchildren	Self-report questionnaire	70 F, 70 M	13.6 (1.3) and 17.1 (0.6)	IV	1	1	1	0	0	1	1	0	0	1	1	1	1	1	1	11
Proportion of studies with positive score (%)	-		-	-	-	*97.0*	*63.6*	*100*	*33.3*	*6.1*	*81.8*	*42.4*	*33.9*	*42.4*	*93.9*	*90.9*	*97.0*	*97.0*	*93.9*	*66.7*	*Mean (SD): 10.5 (2.0)*

* Key to scoring (Law et al. [36]): all items 1 for yes, 0 for no except biases (0 for yes, 1 for no).

1 = Was the study purpose stated clearly? 2 = Was relevant background literature reviewed? 3 = Was the design appropriate for the study question? 4 = Were there any biases present (minimum response rate of 80% for sample bias, blinding of investigators when physical measure were taken)? 5 = Was sample size justified? 6 = Was the sample described in detail? (had to include the number of participants by gender, age,

and a description of where the cohort was sampled from) 7 = Was informed consent obtained? (if not described, assume no); 8 = Were the outcome measures valid? (if all not described, assume no); 9 = Were the outcome measures reliable? (if all not described, assume no); 10 = Results were reported in terms of statistical significance? 11 = Clinical importance was reported? 12 = Were the statistical analysis methods appropriate? 13 = Conclusions were appropriate given the study methods? 14 = Are there implications for clinical practice given the results of the study (based on the experience of the reviewers)? 15 = Were limitations of the study acknowledged and described by the authors?

M = males, F = females
