# Supplementary material for: Thoracic spine pain in the general population: Prevalence, incidence and associated factors in children, adolescents and adults. A systematic review
Source: BMC Musculoskelet Disord. 2009 Jun 29;10:77. doi: 10.1186/1471-2474-10-77 (PMC2720379; doi:10.1186/1471-2474-10-77)
Supplement: Additional File 2 — Prevalence (29 studies) and incidence (5 studies) data grouped by pain definition and reported by age. The data provided describe the prevalence and incidence of TSP across the included studies. [file 1471-2474-10-77-S2.pdf]

Additional File 2: Prevalence (29 studies) and incidence (5 studies) data grouped by pain definition and reported by age.

	Period Prevalence (%)†						Period Incidence (%)†			
Age (yrs)	Point	7 day	1 month	3 month	1 year	Lifetime	1 month	6 month	1 year	25 year
Any back pain										
7-17		10.0 [60]								
8-10			18.7 [57]; 18.5M, 18.5F [11]							
8-12		23.0 [40]								
10-14						17.1M, 18.9F [54]				
10-16		3.4* [59]				12.0* [59]				
11-17	4.0 [53]					15.6 [61]				
12		21.2 [49]	34.8 [49]							
12-18						19.5 [42], 31.2* [42]				
13		2.8M, 6.7F [12]	22.0M, 23.0F [67]							
14		5.6M,							23.5M,	

		11.7F [12]			33.3F [12]
14-16			18.0 [57]; 21.9M, 15.8F [11]		
15		5.6M, 11.7F [12]			11.8M, 33.3F [12]
> 15		0.9-3.6M, 2.9-9.4F ‡ [56]			
16		9.7M, 15.0F [12]			35.3M, 26.7F [12]
17		15.3M, 16.7F [12]			29.4M, 6.7F [12]
20-72		0.5, 0.7* [52]		19.3-27.5 [68]	
35-70				17.0 [13]; 15.0 [46]; 18.0M, 22.0F # [47]	
Pain associated with backpack use					
8-18	6.0, 18.3^				

	[48]			
9	14.0M, 9.0F # [65]			
10	38.0M, 72.0F # [65]			
11	14.0M, 30.0F # [65]			
12	26.0M, 37.0F # [65]			
13	11.7M, 38.0F # [65]	22.9M, 51.4F [58]		
15	31.4 [44]; 24.0M, 41.0F # [65]			
17		31.4M, 40.0F [58]		
Pain interfering with school or leisure				
10			9.3M, 3.5F [45]	
14			9.7M, 9.5F [45]	

16				8.7M, 4.2F [45]				
Pain duration ≥ 1 day								
12		18.0 [50]						
Daily pain for duration ≥ 1 week								
>18				21.5 [51]				9.8 [62]
Pain duration ≥ 15 days								
20-80					0.0-0.6M, 0.3-1.0F [43]			
Pain duration ≥ 1 month								
9-11			4.8M, 7.0F # [66]					
Pain frequency ≥ 1/week								
10							3.8 # [41]	
13						10.3 [69]	7.1 [69]	
Pain associated with low back pain occurring ≥ 1/month								
14-15	20.4M,							

	38.3F [64]			
Frequent pain				
40-69		1.4-6.8 # [55]		
Pain after work				
16-65			34.8 [39]	

* thoracolumbar spinal pain

^ cervicothoracic spinal pain

† M = males, F = females, C = genders combined

‡ age standardised prevalence values

# data extracted from a Figure
