# Supplementary material for: Thoracic spine pain in the general population: Prevalence, incidence and associated factors in children, adolescents and adults. A systematic review
Source: BMC Musculoskelet Disord. 2009 Jun 29;10:77. doi: 10.1186/1471-2474-10-77 (PMC2720379; doi:10.1186/1471-2474-10-77)
Supplement: Additional File 3 — Associated (cross-sectional studies) and risk (prospective studies) factors for TSP grouped according to biopsychosocial categories. Estimates are expressed as an odds ratio (95% CI) unless indicated otherwise. An OR > 1 refers to a positive association with TSP, while and OR < 1 refers to a negative association. Where correlation coefficients are reported, positive values represent a positive association with TSP, while negative values represent a negative association. For statistical tests which do not test for association, a significant outcome (p < 0.05) refers to the factor being greater among individuals with TSP. The data provided summarise factors associated with, and risk factors for TSP. [file 1471-2474-10-77-S3.pdf]

Additional File 3 Associated (cross-sectional studies) and risk (prospective studies) factors for TSP grouped according to biopsychosocial categories. Estimates are expressed as an odds ratio (95% CI) unless indicated otherwise. An OR>1 refers to a positive association with TSP, while and OR<1 refers to a negative association. Where correlation coefficients are reported, positive values represent a positive association with TSP, while negative values represent a negative association. For statistical tests which do not test for association, a significant outcome (p<0.05) refers to the factor being greater among individuals with TSP.

Factor	Association estimate	Risk estimate
	Concurrent musculoskeletal pain	
§ Concurrent neck pain	2.3 (1.5-3.5) [13]; chi square analysis (p<0.05) [48]; r=0.32 [66]	
§ Concurrent low back pain	2.9 (1.8-4.5) [13]; r=0.34 [66]	
§ Concurrent upper limb pain	r=0.25 [66]	
§ Concurrent chest pain	r=0.25 [66]	
§ Concurrent lower limb pain	r=0.21 [66]	
§ Concurrent buttock pain	r=0.22 [66]	
§ Headache in last month	2.9-5.2 (1.4-10.5) [50]	
§ Previous treatment for musculoskeletal pain	2.4 (1.5-3.7) [50]	
	Growth and physical factors	
Puberty (Tanner II)*	1.4 (0.7-2.8) [57]	
Puberty (Tanner III)*	1.6 (0.5-5.4) [57]	

Puberty (Tanner IV)*	1.0 (0.5-2.1) [57]	
Puberty (Tanner V)*	1.1 (0.6-2.3) [57]	
§ Age	0.7 (0.4-1.3) [40]; 1.1 (0.9-1.4) [44]; r=0.009 [65]	6.3 (1.2-43.0) boys [12]; 2.8 (0.7-11.4) girls [12]
§ Gender (female v male)	2.2 (0.9-5.4) [40]; 6.1 (2.2-16.9) [44]; r= -0.21 [65]	
Height	r= -0.03 [65]	
Weight	r= -0.01 [65]	
Body mass index	0.9 (0.8-1.0) [44]; r= -0.002 [65]	
Functional stability (Matthias Test)	1.7 (1.0-2.8) [40]	
§ Difficulty with daily movements (mobility score)	r=0.12 [55]	
Lifestyle and social factors		
§ German speaking	0.2 (0.1-0.5) [40]	
Sports participation (not playing vs. playing)	0.5 (0.2-1.0) [44]; 23.0% vs. 23.0% (p>0.05) [67]	
Hours per week of participation of sport activity in general	No significant difference reported (data not reported) [67]	
§ Participation in a specific sport vs no sport at all (difference in proportion with and without TSP as well as OR [95% CI] where logistic	Jump gymnastics (p=0.6), rhythmic gymnastics (p=0.4), soccer (p=0.9), other ball games (p=0.8), swimming (p=0.6), badminton/tennis (p=0.3), horse riding (p=0.7), running (p=0.9), cycling (p=0.8), roller skating/skate boarding	

regression significant)	(p=0.02; OR 3.1 [1.18-8.08]), martial arts (p=0.5), others (p=0.7) [67]	
§ Participation in a specific sport vs all other sports (difference in proportion with and without TSP as well as OR [95% CI] where logistic regression significant)	Jump gymnastics (p=0.6), rhythmic gymnastics (p=0.5), soccer (p=0.5), other ball games (p=0.8), swimming (p=0.4), badminton/tennis (p=0.09), horse riding (p=0.5), running (p=0.9), cycling (p=0.6), roller skating/skate boarding (p=0.001; OR 3.5 [1.58-7.64]), martial arts (p=0.6), others (p=0.7) [67]	
Transport to and from school on foot or by car vs. other means (bus, bicycle)	1.4 (0.7-3.1) [44]	
Leisure physical activity between individuals with and without pain (mean MET index) ^	F=1.5 (p=0.22) [45]	
Weekly physical activity (sum METs per week) **		1.0 (1.0-1.0) [69]
Musical instrument (playing vs. not) **		1.0 (0.5-2.4) [69]
Working (working vs. not) **		1.3 (0.7-2.5) [69]
Factors related to backpack use		
§ Carrying backpack on two vs. one shoulder	0.4 (0.2-0.9) [44]	
§ Backpack weight	1.0 (0.7-1.3) [44]; r=0.16 [65]	
§ Backpack weight as proportion of body weight	Univariate regression analysis (β=0.05, p<0.05) [48]; r=0.09 [65]	
§ Backpack weight as proportion of body height	r=0.15 [65]	
Duration of backpack carrying	1.0 (0.9-1.0) [44]	

Backpack weight 1.9-2.5 kg †	1.0 (0.5-2.21) [50]
Backpack weight 2.6-3.3 kg †	0.7 (0.4-1.5) [50]
§ Backpack weight 3.4-4.4 kg †	2.2 (1.2-4.3) [50]
Backpack weight 4.5-9.4 kg †	1.2 (0.6-2.3) [50]
Postural factors	
Kyphosis with backpack	1.0 (1.0-1.1) [44]
Kyphosis without backpack	1.0 (1.0-1.1) [44]; r=0.04 [65]; chi square = 0.16 (p=0.98) [62]
Kyphosis difference (with – without backpack)	1.0 (0.9-1.1) [44]
Lordosis with backpack	1.0 (1.0-1.1) [44]
§ Lordosis without backpack	1.1 (1.0-1.2) [44]; r=0.01 [65]; chi square = 2.17 (p=0.54) [62]
§ Lordosis difference (with – without backpack)	0.9 (0.8-1.0) [44]
Craniocervical angle with backpack	1.0 (0.9-1.1) [44]
Craniocervical angle without backpack	1.0 (0.9-1.1) [44]
Craniocervical angle difference (with – without backpack)	1.0 (0.9-1.1) [44]
Biacromial line without backpack (degrees of shoulder level shift)	1.3 (0.9-1.9) [44]
§ Biacromial line with backpack (degrees of shoulder level shift)	1.3 (1.0-1.5) [44]
Biacromial inclination difference (with –	1.2 (1.0-1.4) [44]

without backpack)	
Coronal trunk inclination without backpack	1.6 (0.6-4.2) [44]; r=0.009 [65]; chi square = 0.009 (p=0.96) [62]
§ Coronal trunk inclination with backpack	2.3 (1.2-4.3) [44]
§ Coronal trunk inclination difference (with – without backpack)	2.1 (1.1-4.0) [44]
Sagittal trunk inclination without backpack	1.8 (1.0-3.2) [44]
Sagittal trunk inclination with backpack	1.0 (0.6-1.8) [44]
Sagittal trunk inclination difference (with – without backpack)	0.7 (0.5-1.1) [44]
Pelvic tilt	Chi square = 1.49 (p=0.48) [62]
§ Low number of trunk flexion movements while sitting	Mann Whitney U-test (p<0.05) [49]
Having back twisted (0-10 min vs. no)	1.6 (0.9-2.6) [50]
§ Having back twisted (> 10 min vs. no)	2.2 (1.2-3.8) [50]
**Psychological factors**	
§ Difficulty with homework (sometimes vs. no)	3.5 (1.5-8.1) [50]
§ Difficulty with homework (yes vs. no)	4.3 (1.5-12.6) [50]
§ 5 item mental health index **	1.4 (1.2-1.9) [69]
**Environmental factors**	
Self assessed chair height too high ‡	0.7 (0.2-2.5) [50]
§ Self assessed chair height too low ‡	2.0 (1.1-3.4) [50]

Self assessed chair height don't know	1.6 (0.9-2.8) [50]

* reference = Tanner I

† reference = 0-1.8kg

‡ reference = correct chair height

§ statistically significant factor ($p<0.05$)

^ adjusted for age and gender

** adjusted for growth spurt, initial body height, BMI, age, gender, smoking
